# Learning Curve Analysis of Different Stages of Robotic-Assisted Laparoscopic Hysterectomy

**DOI:** 10.1155/2017/1827913

**Published:** 2017-03-08

**Authors:** Feng-Hsiang Tang, Eing-Mei Tsai

**Affiliations:** ^1^Graduate Institute of Clinical Medicine, School of Medicine, College of Medicine, Kaohsiung Medical University, Kaohsiung, Taiwan; ^2^Department of Obstetrics and Gynecology, Kaohsiung Medical University Hospital, Kaohsiung, Taiwan; ^3^Graduate Institute of Medicine, School of Medicine, College of Medicine, Kaohsiung Medical University, Kaohsiung, Taiwan

## Abstract

*Objective*. To analyze the learning curves of the different stages of robotic-assisted laparoscopic hysterectomy.* Design*. Retrospective analysis.* Design Classification*. Canadian Task Force classification II-2.* Setting*. Kaohsiung Medical University Hospital, Kaohsiung, Taiwan.* Patient Intervention*. Women receiving robotic-assisted total and subtotal laparoscopic hysterectomies for benign conditions from May 1, 2013, to August 31, 2015.* Measurements and Main Results*. The mean age, body mass index (BMI), and uterine weight were 46.44 ± 5.31 years, 23.97 ± 4.75 kg/m^2^, and 435.48 ± 250.62 g, respectively. The most rapid learning curve was obtained for the main surgery console stage; eight experiences were required to achieve duration stability, and the time spent in this stage did not violate the control rules. The docking stage required 14 experiences to achieve duration stability, and the suture stage was the most difficult to master, requiring 26 experiences. BMI did not considerably affect the duration of the three stages. The uterine weight and the presence of adhesion did not substantially affect the main surgery console time.* Conclusion*. Different stages of robotic-assisted laparoscopic hysterectomy have different learning curves. The main surgery console stage has the most rapid learning curve, whereas the suture stage has the slowest learning curve.

## 1. Introduction

Robotic-assisted laparoscopic surgery in gynecology has gained popularity and has been applied to many types of gynecological surgeries since its approval by the US Food and Drug Administration in 2005 [1]. Through the high-definition, three-dimensional image displayed on the surgeon's console, the surgeon can control the robotic arms to perform the surgery [2]. From the patient's perspective, robotic-assisted laparoscopic surgery is as advantageous as other minimally invasive surgeries. From the surgeon's perspective, robotic surgery typically has a more rapid learning curve, facilitates intracorporeal suturing and knot-tying, and is more suitable for highly complicated procedures that require extensive dissection and appropriate anatomical restoration than conventional laparoscopic surgery [3–5]. A robotic platform is the logical step forward from laparoscopy, and if cost considerations are not addressed, it may become a popular surgical technique among gynecologists worldwide [1].

The entire laparoscopic procedure can be divided into three stages: (1) inserting the trocars and preparing the video telescope and laparoscopic instruments; (2) performing the main surgery; and (3) removing the specimens and restoring the anatomy. Although robotic surgery is similar to conventional laparoscopic surgery, two major differences exist. First, robotic surgery requires the docking of the video telescope and laparoscopic instruments on the robotic arms before the initiation of the main surgery; second, the surgeon controls the robotic arms to perform the main surgery and to restore the anatomy through the console machine.

Most studies on the learning curve of robotic surgery have evaluated the entire operation time. During the past two years, some studies have analyzed the different stages of robotic surgery; however, they analyzed only one of these stages [6, 7]. Therefore, the present study performed a stage-by-stage analysis of the learning curve for robotic-assisted laparoscopic hysterectomy to clearly understand the different stages. Because the uterus removal procedure is similar between robotic surgery and conventional laparoscopic surgery, this stage was not analyzed. Only the docking, main surgery console, and suture stages were analyzed in the present study. Furthermore, we examined the possible effects of three factors, namely, patient body mass index (BMI), uterine weight, and presence of adhesion, on the different stages.

## 2. Materials and Methods

In this study, we reviewed all clinical records of patients who underwent robotic-assisted total and subtotal laparoscopic hysterectomies for benign conditions from May 1, 2013, to August 31, 2015, performed by a single senior laparoscopic gynecologist at Kaohsiung Medical University Hospital, because other doctors performed only a few robotic-assisted gynecological surgeries. Patients who underwent adnexal surgery or other procedures at the same operation were excluded. A total of 43 cases were included in the present study. The time spent in each stage was recorded by the circulating nurse at operation room. The uterine weight was calculated immediately after uterine removal.

The docking time was calculated as the time between the completion of trocar insertions and the docking of the video telescope and two robotic arms. The four trocars consisted of a central 12 mm wide trocar for the telescope, two bilateral 7 mm wide trocars for the two robotic arms, and a 5–12 mm wide accessory trocar. The position of the four trocars depended on the specimen size. Generally, the central 12 mm trocar was located at the umbilicus, and the 7 mm trocars, one on either side, were 12 cm lateral and 2 cm downward to the central trocar. For a large uterus, with a fundus–umbilicus distance of <10 cm, the central trocar was placed at least 10 cm above the uterine fundus. The accessory trocar was inserted midline between the central telescope and the left-side 7 mm trocar, when required.

The main surgery console time was defined as the time taken to perform the main surgery. Conventionally, this includes the time of the main surgery and anatomical restoration. However, in this study, only the time taken for the main surgery was calculated; the time of anatomical restoration was calculated as a part of the suture stage to clearly identify the different stages of robotic surgery. All procedures were performed using robotic-assisted laparoscopic techniques. The endpoint of the main surgery console stage of total hysterectomy was the time at which the uterus was completely separated from the vagina, and the endpoint of subtotal hysterectomy was the separation of the uterine body from the cervix. A conventional uterine manipulator was used in the surgery, and vaginal gauze was inserted to prevent CO2 escape after the vagina was opened.

In the total hysterectomy group, the time taken to close the vaginal cuff by using barbed sutures was defined as the time of the suture stage. In the subtotal hysterectomy group, the time required to reperitonize the uterine cervix was considered the time of the suture stage (Figure 3).

We used a quality control chart to determine the number of experiences required by an experienced laparoscopic gynecologist to achieve performance stability for the three different stages of robotic surgery. The quality control chart was first used in the 1920s in Bell Lab and has since been widely applied in the industry to monitor product quality. If a product violates the control rules, it would be eliminated [8]. The average time of each stage was used as the standard. The control rules, which determined that the time exceeded the standard time, included (1) data points that were more than three standard errors above the average, (2) the last two of the three consecutive values above two standard errors, and (3) the last four of the five consecutive values above one standard error.

All data were compared with the standard values. The data which are against the control rules will be violated. The first case number after the last violated case number was considered the least number of experiences required to achieve stability, indicating that the minimum case numbers are needed in the stage and did not violate the control rules.

In addition, we investigated the possible effects of patient BMI, uterine weight, and the presence of adhesion on the different stages of robotic surgery. BMI was calculated as the body mass (kg) was divided by the square of the body height (m). The uterine weight (g) was obtained immediately after uterus removal, and the presence or absence of adhesion was determined based on the operation records. The effects of BMI and uterine weight were evaluated through analysis of variance, and the effect of the presence of adhesion was analyzed through a *t*-test. All statistical analyses were performed using IBM SPSS Statistics 22.0 (Chicago, IL).

## 3. Results

A total of 43 robotic-assisted laparoscopic hysterectomies (subtotal = 6; total = 37) for benign conditions were performed from May 1, 2013, to August 31, 2015, by a single senior laparoscopic gynecologist.  Table 1 presents the baseline demographic data. The mean age, BMI, and uterine weight were 46.44 ± 5.31 years, 23.97 ± 4.75 kg/m^2^, and 435.48 ± 250.62 g, respectively.

First, we investigated the potential effects of BMI, presence of adhesion, and uterine weight on the different stages of robotic surgery. BMI did not substantially affect the three stages. The effects of the presence of adhesion and uterine weight were evaluated only for the main surgery console stage, because we assumed that these factors only affected this stage. The presence of adhesion and uterine weight did not substantially affect the main surgery console time (Table 2).

**Figure 1:**
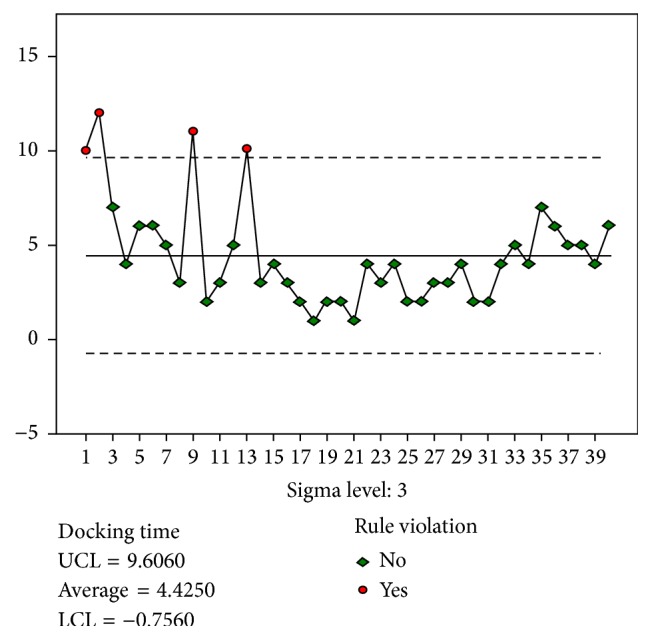
Control chart for Docking time.

**Figure 2:**
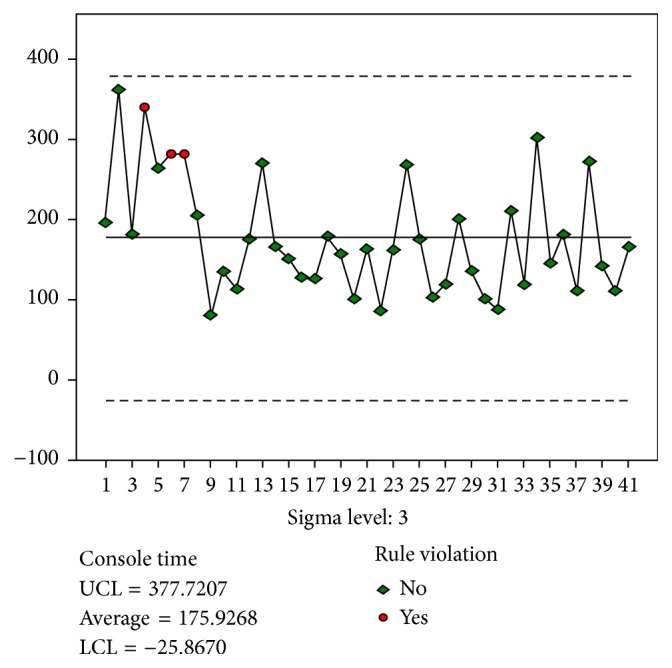
Control chart for Main Surgery Console time.

**Figure 3:**
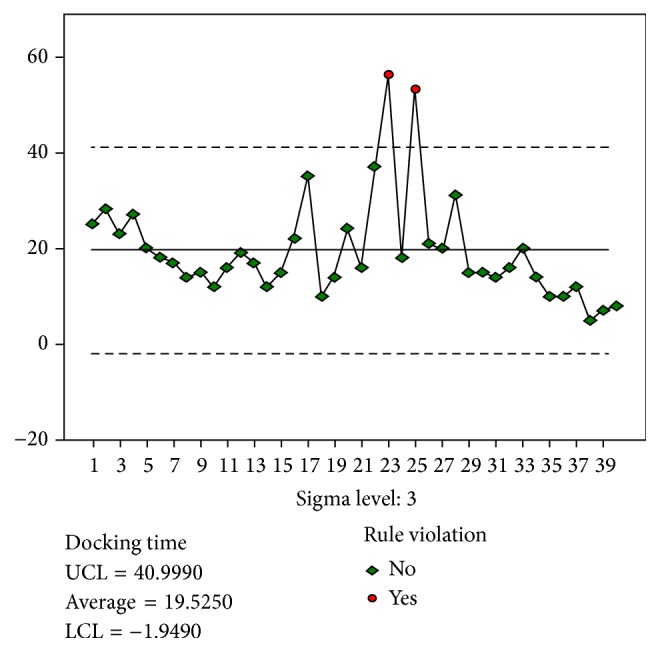
Control chart for Suture time.

The results for the docking stage were analyzed based on the quality control chart (Figure 1). A total of 14 and 8 experiences were required to achieve stability in the docking and main surgery console stages (Figure 2), respectively. However, 26 experiences were required to achieve stability in the suture stage.

## 4. Discussion

In our study, BMI did not markedly affect the time spent in the docking, main surgery console, and suture stages, consistent with other studies [9–11]. In addition, the presence of adhesion did not substantially affect the main surgery console time, which may be because robotic surgery facilitates delicate dissection through wrist-simulating instruments. Furthermore, the uterine weight did not substantially affect the main surgery console time. Although a previous study on laparoscopic hysterectomy reported a positive correlation between the uterine weight and operation time [12], a recent study by Silasi et al. [13] demonstrated that increasing uterine size does not proportionally affect the operation time in robotic or abdominal hysterectomy. In minimally invasive hysterectomies, most surgeons have to morcellate the large uterus into small pieces to facilitate its removal from the abdominal cavity, which is typically time consuming. In our study, we only considered the time required by the surgeon to perform the main surgery using the console machine and excluded the time required for uterus removal. Therefore, the time required for uterus morcellation was not included, which may have affected the study findings. In addition, these findings suggest that the uterine weight considerably affects time-consuming uterus morcellation and removal procedures, thus consequently affecting the total operation time.

**Figure 4:**
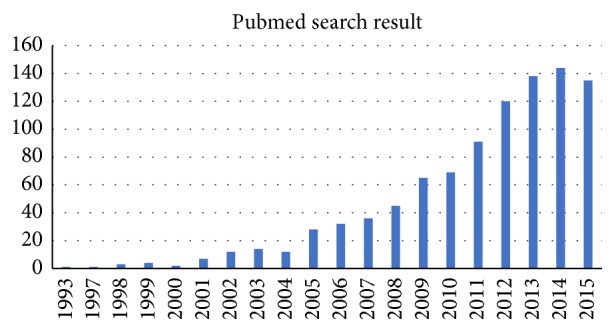
The number of citations from a PubMed search with the keyword “learning curve robotic” published before 2015.

Learning curve analyses of robotic surgery have gained popularity in recent years (Figure 4). However, a consensus on the analysis method is yet to be reached. Three types of methods are commonly used, namely, the chronological grouping method, power-law curve analysis, and cumulative sum analysis. In the chronological grouping method [6, 14], the study groups are chronologically categorized into subgroups, and subsequent subgroup analyses are conducted. However, the subgrouping method is arbitrary, and different grouping methods may provide variable results. More subgroups may predict the turning point more effectively, which represents the lowest case numbers or the shortest time required to learn a new technique, of the learning curve for a new technology; however, they may also reduce the reliability and validity of the analyses results because of the reduced size of each subgroup. In the power-law curve analysis [7], the relationship between the reduced operation time and the increased case numbers is assumed to follow a power-law distribution. However, the operator's stability cannot be evaluated from the regression curve, and the turning point may have advanced without achieving stability. Cumulative sum analysis is frequently used to evaluate stability for quality control in the industry. Several studies have used this method to analyze learning curves [15–17] because it can rapidly detect the changes in stability. However, for an accurate cumulative sum analysis, the standard value should be known before analysis, which is unlikely in learning curve analysis of a new technique. Most studies using the cumulative sum analysis method have used the personal mean operative time as the standard. Because the operation time is initially longer and highly variable, the cumulative sums increase rapidly, and the experiences required to remain within the control limits are longer even if the time spent by the surgeon has achieved stability.

Therefore, we used the quality control chart for learning curve analysis. The mean time spent by the gynecologist was considered to be the standard. All data were compared with the standard values, and the control rules were used to determine the values that indicated instability (i.e., values that violated the control rules). This method enables the determination of the least number of experiences required for a gynecologist to achieve stability and the exact case number before the violated case number. However, the quality control chart is prepared by continuously examining the industry trends, and the purpose is to exclude the product not meeting the quality standards. Therefore, the appropriateness of this method requires further research.

Until recently, the learning curves of new surgical techniques have typically been assessed using the entire operation time. Because elements of robotic surgery are similar to those of conventional laparoscopic surgery, we eliminated similar elements and analyzed only the distinctive elements of robotic surgery to enable the highly accurate determination of the learning curve for an experienced laparoscopic gynecologist. Of the three stages, the main surgery console stage had the most rapid learning curve, followed by the docking and suture stages, because an experienced laparoscopist is most familiar with the main surgery console stage, and only the method of instrument control has to be learnt.

The docking stage substantially differs from conventional laparoscopic surgery; therefore, teamwork is required. Thus, more experiences are required to achieve stability in the docking stage than in the main surgery console stage, which involves only one or two persons.

The suture stage is typically regarded as the most difficult and technically demanding stage of conventional laparoscopic surgery; however, only a few objective studies on this stage have been conducted. Our study revealed that the suture stage requires the most number of experiences to achieve stability.

The present study results validated our initial assumption that the learning curves vary across the different stages of robotic surgery. Thus, we suggest that the different stages of the procedure should be evaluated while determining the learning curve of robotic surgery. Furthermore, a consensus on the appropriate research method for learning curve analysis is yet to be reached. A standard method is required for effective analyses, comparisons across analyses, and result interpretations for such studies. Moreover, a comparative study on the different methods of learning curve analysis is warranted.

The present findings revealed that suture stage is the most difficult stage to master; therefore, we suggest that more suturing practice on a simulator would be beneficial in a doctor training program on robotic surgery. Furthermore, a large-scale and comprehensive study is required to thoroughly understand the learning curve of robotic surgery.

## Figures and Tables

**Table 1:** Demographic data for individuals undergoing robotic hysterectomy surgery.

Hysterectomy type		Age (years)	Height (cm)	Weight (kg)	BMI (kg/m^2^)	Specimen weight (g)
Total	Subtotal					
37	6	46.44 ± 5.31	158.1 ± 1.65	59.6 ± 11.26	23.97 ± 4.75	435.48 ± 250.62

**Table 2:** The influence of BMI, adhesion, and specimen weight on docking time, main surgery console time, and suture time.

Factors	Docking stage (minutes)	Main surgery console stage (minutes)	Suture stage (minutes)
BMI (kg/m^2^) (*N*)			
BMI < 20 (7)	4.00 ± 3.46	152.57 ± 45.16	19.43 ± 15.66
20 < BMI < 24 (18)	4.76 ± 2.82	176.83 ± 81.70	18.05 ± 6.63
24 < BMI < 27 (10)	4.00 ± 1.69	177.88 ± 70.92	19.63 ± 9.59
BMI > 27 (7)	4.50 ± 2.67	192.38 ± 77.19	22.63 ± 15.10
	*P* = .950	*P* = .733	*P* = .860
Presence of adhesions (*N*)			
Yes (19)		175.68 ± 68.39	
No (22)		176.14 ± 76.98	
		*P* = .984	
Uterine weight (SW), (g) (*N*)			
SW < 250 g (11)		175.27 ± 78.88	
250 g ≤ SW < 500 g (16)		171.06 ± 70.47	
500 g ≤ SW < 750 g (9)		171.56 ± 80.56	
SW ≥ 750 g (5)		200.80 ± 64.00	
		*P* = .876	

Data are presented as the mean ± standard error (*N* = [number of cases OR numbers]).
